# PLETHORA transcription factors promote early embryo development through induction of meristematic potential

**DOI:** 10.1242/dev.202527

**Published:** 2024-06-17

**Authors:** Merijn Kerstens, Carla Galinha, Hugo Hofhuis, Michael Nodine, Renan Pardal, Ben Scheres, Viola Willemsen

**Affiliations:** ^1^ Cluster of Plant Developmental Biology, Cell and Developmental Biology, Wageningen University & Research, Droevendaalsesteeg 1, 6708 PB Wageningen, The Netherlands; ^2^ Department of Molecular Genetics, Utrecht University, Padualaan 8, 3584 CH Utrecht, The Netherlands

**Keywords:** Embryogenesis, Meristem, Plant development, Stem cell

## Abstract

Plants are dependent on divisions of stem cells to establish cell lineages required for growth. During embryogenesis, early division products are considered to be stem cells, whereas during post-embryonic development, stem cells are present in meristems at the root and shoot apex. PLETHORA/AINTEGUMENTA-LIKE (PLT/AIL) transcription factors are regulators of post-embryonic meristem function and are required to maintain stem cell pools. Despite the parallels between embryonic and post-embryonic stem cells, the role of PLTs during early embryogenesis has not been thoroughly investigated. Here, we demonstrate that the PLT regulome in the zygote, and apical and basal cells is in strong congruence with that of post-embryonic meristematic cells. We reveal that out of all six PLTs, only *PLT2* and *PLT4/BABY BOOM* (*BBM*) are expressed in the zygote, and that these two factors are essential for progression of embryogenesis beyond the zygote stage and first divisions. Finally, we show that other PLTs can rescue *plt2 bbm* defects when expressed from the *PLT2* and *BBM* promoters, establishing upstream regulation as a key factor in early embryogenesis. Our data indicate that generic PLT factors facilitate early embryo development in *Arabidopsis* by induction of meristematic potential.

## Teaser

**Summary:** PLETHORA transcription factors induce meristematic potential during early *Arabidopsis* embryogenesis, which is in strong congruence with their function in maintenance of post-embryonic meristems.

## INTRODUCTION

In plants and animals, the products of the first rounds of embryonic divisions are thought to be totipotent or pluripotent stem cells that are able to differentiate into most cell lineages – if not all – that constitute an organism. After the establishment of the initial body plan by the progeny of these cells, divisions of self-renewing stem cells produce new cells to generate, maintain or repair organ systems. Plants show extensive post-embryonic development and require stem cells for continuous growth of the root and shoot apices throughout the entire life cycle. Root and shoot stem cells are located in specialized tissue types called meristems and form defined layers through precisely controlled divisions and division directions. In many species, including *Arabidopsis*, embryogenesis starts with zygote expansion before an asymmetric division that forms a one-cell-stage embryo consisting of an apical cell and a basal cell. Whereas the apical lineage eventually gives rise to the embryo proper, the basal lineage is restricted to the quiescent center, root cap precursor cells and the transient suspensor. The apical and basal cells are thus progenitors of future differentiated tissues generated through a continuous series of tightly controlled divisions. In that regard, they are comparable to the stem cells in post-embryonic meristems. However, the extent to which their analogous functions have a common mechanistic basis, has been unclear to date.

In plant meristems, PLETHORA/AINTEGUMENTA-LIKE (PLT/AIL) transcription factors (TFs) of the APETALA2/ETHYLENE-RESPONSIVE ELEMENT BINDING PROTEIN family are key post-embryonic developmental patterning factors (Horstman et al., 2014; Scheres and Krizek, 2018). In the root meristem, PLT1, PLT2, PLT3 and BABY BOOM (BBM, also known as PLT4) indirectly read out an auxin gradient to establish stable tissue zonation by controlling cell division and differentiation (Mahonen et al., 2014; Santuari et al., 2016; Roszak et al., 2021) and they are required for stem cell maintenance (Aida et al., 2004; Blilou et al., 2005). *plt1 plt2* double mutants lose root meristem identity after 6-8 days, whereas *plt1 plt2 plt3* triple mutants fail to develop root systems entirely (Aida et al., 2004; Galinha et al., 2007). In the shoot apical meristem, PLT3, PLT5 and PLT7 establish spiral positioning of flower primordia in a PIN1-dependent manner and loss of all three factors results in aberrant phyllotaxis (Prasad et al., 2011; Pinon et al., 2013). In lateral roots, these three factors act redundantly to position primordia and to maintain primordium (Hofhuis et al., 2013; Du and Scheres, 2017; Kerstens et al., 2021). Furthermore, PLT1 and PLT2 interact with WOX5 or WOX7 to induce endogenous auxin biosynthesis and confer pluripotency to the callus and, together with PLT3, PLT5 and PLT7, activate *de novo* shoot regeneration pathways in root calli (Kareem et al., 2015; Zhai and Xu, 2021). PLTs appear to be required during the early stages of embryogenesis as well. We previously reported that homozygous combinations of *plt2* and *bbm* alleles could not be recovered from crosses of higher-order *plt* mutants (Galinha et al., 2007). In line with this observation, it was recently demonstrated that *plt2 bbm* mutants arrest early in embryogenesis and display morphological defects, and even *plt2* and *bbm* single-mutant embryos show phenotypes, i.e. they develop faster than wild type (Chen et al., 2022). Moreover, weak *BBM* expression was detected in ∼2% of ovules, and consistently in one- and two-cell-stage embryos (Chen et al., 2022), but the zygotic/embryonic expression pattern of *PLT2* (and other PLTs) remains unknown. Ectopic overexpression of PLTs promotes the production of embryos from vegetative tissues (i.e. somatic embryogenesis) (Boutilier et al., 2002; Tsuwamoto et al., 2010; Horstman et al., 2017). In addition, induction of *BBM*-like genes in the egg cell can trigger asexual embryogenesis (El Ouakfaoui et al., 2010; Conner et al., 2015; Conner et al., 2017).

Although PLTs are intricately connected to meristematic function and cell pluripotency, it remains unknown whether they perform similar roles during early embryogenesis. We therefore investigated which PLTs are expressed in the zygote, and apical and basal cells, and the extent to which they confer meristematic potential to these cells through activation of direct target genes shared with apical meristems. We show that generic PLT activity in the native expression domains of *PLT2* and *BBM* activates a meristem-like transcriptional network and is essential for progression beyond the zygote stage. Therefore, the role of PLT TFs is likely to activate a generic meristematic stem cell program from early embryogenesis onward.

## RESULTS

### The PLT regulome during early embryogenesis is meristem-like

Given that PLTs are key TFs in the maintenance of stem cell niches, we hypothesized that they confer meristematic properties to the early embryonic cells, from which all future cell types arise. To investigate this notion, we examined the degree to which downstream PLT targets in apical meristems overlap with those during early embryogenesis. We first selected candidate direct PLT target genes by overlaying PLT chromatin immunoprecipitation followed by sequencing (ChIP-seq) and DNA affinity purification and sequencing (DAP-seq) datasets (Table S9). We extended existing sets with an additional PLT3-DAP experiment on young roots, because previous PLT-DAP data had only low read counts and used leaf material in which PLTs are typically not strongly expressed. We found 2644 significant binding sites that had overlap between at least two datasets and were centrally enriched for the canonical PLT-binding motif (Santuari et al., 2016) corresponding to 2406 associated genes within a [−4 kb, +4 kb] range (Table S1; Table S9; Fig. S1A-C). To filter this direct target gene list further, we used *PLT* overexpression data from whole seedlings (Santuari et al., 2016) to include only genes consistently upregulated by at least one PLT member, resulting in 334 PLT target genes (Table S1).

**Fig. 1.**
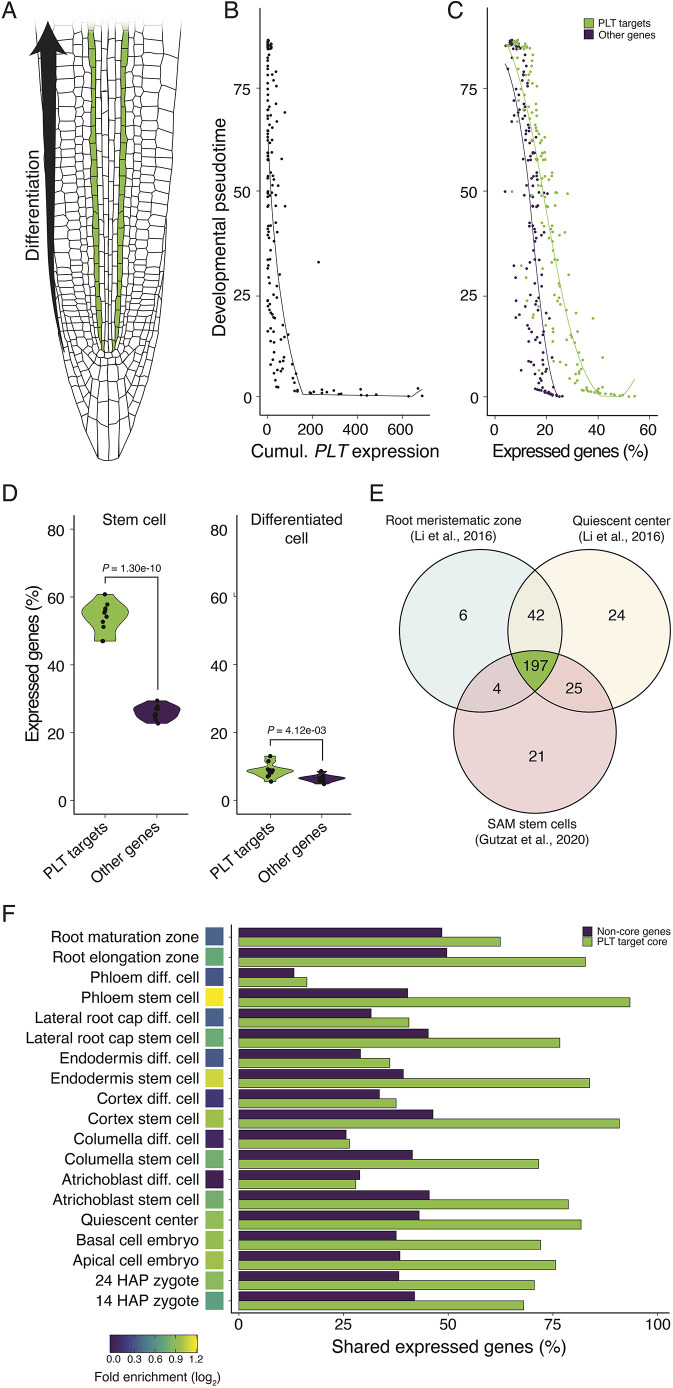
**Expression of directly regulated PLT target genes is enriched in stem(-like) and embryonic cells.** (A) Schematic overview of the protophloem sieve element lineage on the developmental root axis. (B) Cumulative PLT gene expression in the protophloem lineage over developmental pseudotime. The *y*-axis relates to the schematic in A. (C) Percentage of expressed PLT target and non-target genes over pseudotime. Each dot in B and C is the average value of cells in bins of 0.1 pseudotime (*n*>1). Lines are loess regressions. (D) Percentage of expressed PLT target and non-target genes in stem cell-like and differentiated protophloem cells (one-tailed Welch's *t*-test, *n*=10). Shaded areas represent the distribution of data points per group. (E) Venn diagram of expressed PLT target genes in meristematic tissues. (F) Percentage of PLT core targets and non-PLT core targets expressed in early embryonic, stem(-like) and differentiated cells. Heat map displays the log_2_ ratio between the expressed PLT core and non-PLT core genes. Note that the ‘quiescent center’ values in this panel are based on the Shahan et al. (2022) dataset.

To verify our final selection of 334 high-confidence target genes, we took advantage of a high-resolution single-cell RNA sequencing (scRNA-seq) dataset that describes the developmental trajectory of the protophloem sieve element lineage from stem cells towards differentiation, in which PLT2 has been shown to be a central factor (Roszak et al., 2021) (Fig. 1A). Within this lineage, cumulative *PLT* transcript levels peaked at the earliest points in pseudotime and thereafter rapidly decreased (Fig. 1B), in line with the function and localization of PLTs in the root stem cell niche (Aida et al., 2004). We then investigated how the 334 PLT target genes were expressed along the same developmental axis, and whether expression was enriched at early points in pseudotime in comparison with non-PLT target genes (i.e. genes without associated ChIP/DAP peaks and without upregulation through overexpression). We observed a gradual reduction in the percentage of expressed target genes from ∼40% to ∼10% over developmental pseudotime, a much sharper reduction than that of non-PLT target genes, which declined from ∼20% to ∼10% (Fig. 1C). In the ten earliest cells, the percentage of expressed target genes was more than twofold higher than that of non-target genes (*P*=1.3e−10), as opposed to the ten latest cells, where only a minute enrichment could be detected (*P*=4.12e−03; Fig. 1D). We also performed this analysis on the two gene lists that were overlapped to yield the 334 target genes: the PLT-bound and the PLT-upregulated list. The percentage of expressed PLT-bound genes was only weakly enriched in early cells, likely resulting from non-functional binding events picked up through the ChIP or DAP procedures (Fig. S1D,E). The percentage of expressed PLT-upregulated genes, by contrast, was most enriched, which we attributed to the fact that this set also included indirect targets through *PLT* overexpression (Fig. S1D,E).

We next sought to determine whether expression of PLT target genes was also enriched in root cell types other than the protophloem at early developmental pseudotimes. To this end, we analyzed an untargeted scRNA-seq dataset of root tips (Shahan et al., 2022) and first determined the percentage of cells expressing *PLT* genes in various lineages over pseudotime. In the atrichoblast, columella, cortex, endodermis, and lateral root cap lineages, we observed a clear increase in cells expressing PLT genes in early pseudotime groups compared with late groups, and 100% of quiescent center cells expressed PLT genes (Fig. S2A). These findings are consistent with known PLT gene expression domains (Galinha et al., 2007) and in line with PLT gene expression in the protophloem scRNA-seq set. It was noticed that the trichoblast lineage, however, did not express PLT genes even in the earliest time group; for this reason it was not analyzed further. We then repeated the PLT target and non-PLT target expression analysis that was performed for the protophloem lineage for these other root cell types. In all lineages, the earliest time groups consistently showed an enrichment of PLT target gene expression over non-PLT target expression, especially the quiescent center, which was lost in later time groups (Fig. S2B). Moreover, the ten earliest cells of the atrichoblast, columella, lateral root cap, and quiescent center lineages expressed significantly more PLT target genes than non-PLT target genes, in contrast to the ten latest cells, for which no increase could be identified (Fig. S2C,D). We thus conclude that the identified direct target genes are enriched in the PLT gene expression domain of the root meristem.

If direct PLT target genes are predominantly expressed in stem and stem-like cells, then this property must be shared between different meristematic tissues. We gathered expression data of the root meristematic zone (Li et al., 2016), quiescent center (Li et al., 2016) and shoot stem cells (Gutzat et al., 2020), and checked which target genes were expressed in common. We identified a core set of 197 PLT target genes expressed in each of these tissues (Table S2, Fig. 1E). Gene ontology (GO) network analysis revealed that the biological process ontologies formed seven clusters containing terms we could collectively classify as regulation (I), response (II), development and morphogenesis (III), chromatin and cytoskeleton organization (IV), cell division (V), import, transport and localization (VI), and (DNA) metabolism and modification (VII) (Fig. S1F; Tables S3, S4). Many terms within each cluster could be linked to meristem function (e.g. ‘regulation of meristem development’, ‘meristem maintenance’, ‘mitotic cell cycle’, and ‘gene expression’), in line with the expected function of genes shared between different meristematic tissues.

We then investigated the extent to which these PLT ‘core’ target genes were expressed in stem(-like) and differentiated cells across various cell lineages in the single-cell root datasets (Roszak et al., 2021; Shahan et al., 2022). In all analyzed stem cell types, more than 70% of the PLT target core was expressed, roughly a twofold enrichment over non-core genes (Fig. 1F). In contrast, differentiated cells of these lineages did not exhibit clear enrichment of core genes (Fig. 1F). Additionally, the enrichment of PLT core targets over non-core genes was elevated in the root elongation zone in comparison with the root maturation zone (Li et al., 2016), where PLTs are typically still present (Galinha et al., 2007) (Fig. 1F). Next, we investigated how this core set was expressed during early embryogenesis. Strikingly, zygotes at both 14 and 24 hours after pollination (HAP) (Zhao et al., 2019), as well as apical and basal cells (Zhou et al., 2020), expressed between 70 and 75% of PLT core targets (Tables S5-S8), and displayed enrichment over non-core genes to a similar degree as that observed in all root stem cell types (Fig. 1F). We conclude that, during early embryogenesis, the PLT regulome largely resembles that of meristematic cells.

### PLT genes are expressed in partially overlapping domains from the zygote onward

**Fig. 2.**
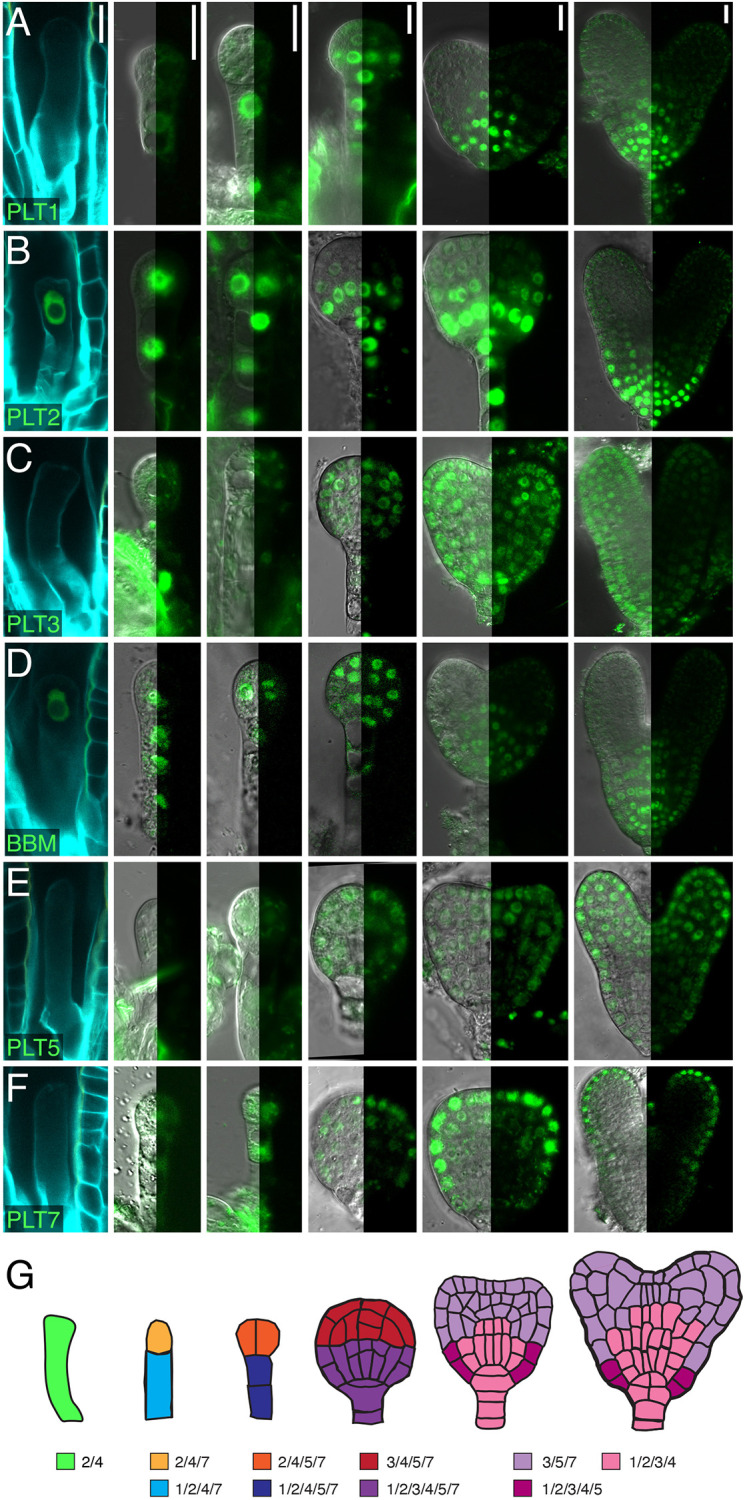
**PLT genes are expressed in partially overlapping expression domains during early and late embryogenesis.** (A-F) Translational pPLT::PLT-YFP reporter signal of PLT1 (A), PLT2 (B), PLT3 (C), BBM (D), PLT5 (E) and PLT7 (F) during embryogenesis. Left to right: 32 HAP zygote, one-cell stage, two- to four-cell stage, globular stage, triangular stage and heart stage. Zygotes were stained with SR2200; DIC and confocal signal are shown for other stages. Scale bars: 10 µm. (G) Schematics of PLT gene expression domains per stage marked with different colors; numbers denote PLT identity (e.g. 1=PLT1, 4=BBM).

Because the zygote, and apical and basal cells exhibit a meristem-like PLT readout, we investigated which PLTs drive this developmental response. We thus characterized expression patterns of the entire PLT gene family throughout embryogenesis by using genomic PLT-YFP translational fusions under the control of their native promoters (pPLT::gPLT-YFP ; Aida et al., 2004; Galinha et al., 2007) from the zygote stage onward. At the onset of embryogenesis, we detected *PLT2* and *BBM* expression in ∼84% (101/120) and ∼74% (26/35) of elongated zygotes (Fig. 2B,D), in contrast to the other PLTs, for which signal was never observed (Fig. 2A,C,E,F). At the one-cell stage, we observed *PLT2*, *BBM* and *PLT7* expression in both the apical and basal cells (Fig. 2B,D,F); *PLT1* was only expressed in the basal cells (Fig. 2A). At the two- to four-cell stage, *PLT1* was restricted to the early suspensor cells (Fig. 2A), whereas *PLT2*, *BBM*, *PLT7* and *PLT5* were expressed, albeit weakly, in both apical and basal lineages (Fig. 2B,D-F). PLT3-YFP could not be detected until the globular stage (Fig. 2C). From this stage onward, all PLT genes were expressed in the embryo proper. In contrast to *PLT3*, *BBM* and *PLT5*, which were expressed similarly in all cells of the globular-stage embryo (Fig. 2C-E), the expression of *PLT1* and *PLT2* was restricted to the lower tier of the embryo proper (Fig. 2A,B), whereas *PLT7* expression was primarily localized in the outer layer (the protoderm) of the proembryo (Fig. 2F). In later stages, expression of *PLT1*, *PLT2*, *PLT3* and *BBM* extended to the quiescent center progenitor lens-shaped cell and were subsequently detected in the quiescent center, columella and vascular tissue (Fig. 2A-D). Simultaneously, *PLT3*, *PLT5* and *PLT7* expression was detected throughout the upper tier, specifically in the protoderm, the inner tissue and the shoot apical meristem initials (Fig. 2C,E,F). At this point, PLT gene expression patterns thus separate according to their future position: a root apical meristem domain formed by *PLT1-4* (Galinha et al., 2007), and a shoot apical meristem domain formed by *PLT3*, *PLT5* and *PLT7* (Prasad et al., 2011). Taken together, PLT gene expression patterns form distinct overlapping domains throughout embryogenesis and are already observed in the earliest embryonic cells (Fig. 2G).

In our expression analysis, we deliberately chose to avoid transcriptional fusions to retain potential regulatory elements in intronic sequences, but this approach still relies on integration of transgenic T-DNAs in non-native genomic positions. To gain insight in the native transcriptional dynamics of PLT genes during early embryogenesis, we mined three RNA-seq datasets of early embryos (Hofmann et al., 2019; Zhao et al., 2019; Zhou et al., 2020). In agreement with our observations from the translational fusions, *PLT2* and *BBM* were strongly expressed in both 14 HAP and 24 HAP elongated zygotes, although *PLT1* and *PLT5* also showed weak expression in the RNA-seq data (Fig. S3A). Moreover, *PLT1*, *PLT2*, *BBM* and *PLT7* were expressed moderately to strongly at the one-cell stage, in contrast to the weakly expressed *PLT3* and *PLT5* (Fig. S3A). In accordance with the analyzed reporter lines, *PLT2*, *BBM* and *PLT7* (and to a very minor degree *PLT1*) were the only PLT genes expressed in the apical cell at this stage, emphasizing that particularly these family members could have potential roles in early embryogenesis (Fig. S3A). From the 32-cell stage onward, all PLT genes were expressed in the embryo proper and suspensor (Fig. S3A), consistent with our translational reporters. The localization patterns observed at the globular stage also largely overlapped with those obtained by single-nucleus mRNA sequencing of globular embryos (Fig. S3B,C). We thus demonstrate that our translational PLT-YFP fusions are in strong congruence with published transcriptome data and that multiple PLT genes are expressed during the early stages of embryo development.

### Only PLT2 and BBM are essential for early embryonic cell progression

**Fig. 3.**
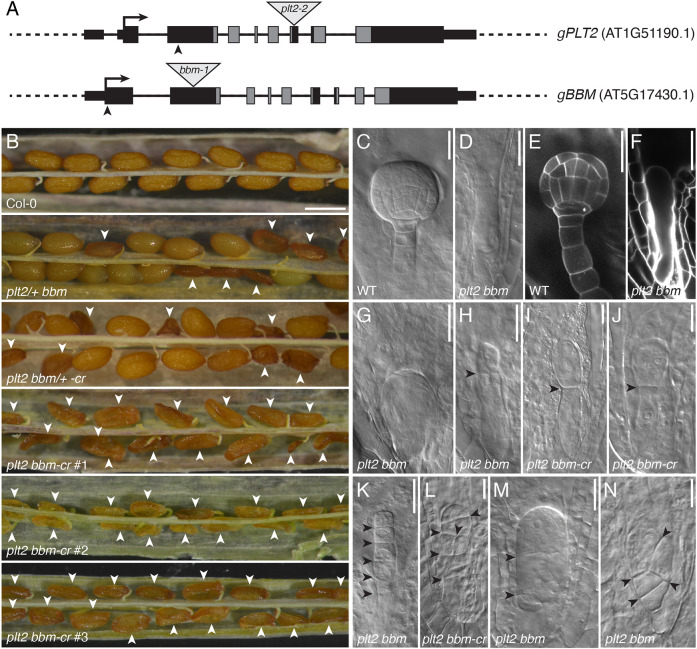
**PLT2 and BBM are required for early embryogenesis.** (A) Gene models of *PLT2* (AT1G51190.1) and *BBM* (AT5G17430.1). Black rectangles and lines indicate exons and introns, respectively. UTRs are denoted by thin rectangles. AP2 domains are marked in gray. Gray triangles denote T-DNA insertion sites. Arrowheads indicate CRISPR PAM sites. (B) Aborted seeds and viable seeds in siliques of Col-0, in self-fertilized *plt2/+ bbm* or *plt2 bbm/+ -cr*, and in double-homozygous *plt2 bbm-cr* T_1_ lines. Arrowheads point to aborted seeds. (C-N) Phenotypic diversity of *plt2 bbm* and *plt2 bbm-cr* embryos at similar stages of development compared with the wild type (C,E), including arrests at the zygote stage (D,F,G), at the one-cell stage (H-J), and after erratic divisions (K-N). *plt2 bbm* T-DNA mutants are shown in D,F-H,K,M,N; *plt2 bbm-cr* mutants in I,J,L. C,D,G-N are DIC images; E,F are confocal images. Arrowheads indicate the location and direction of division planes. Scale bars: 500 µm (B); 25 µm (C-N).

Given that in root meristems (Galinha et al., 2007) and shoot meristems (Prasad et al., 2011), PLTs act redundantly through overlapping expression domains, similar functional redundancy might apply to PLT genes expressed in early embryos. As *PLT2* and *BBM* were the primary PLT genes expressed in the zygote, and *PLT2*, *BBM* and *PLT7* were the only ones expressed in both the apical and basal cells in one-cell-stage embryos, we generated double-mutant combinations to inspect early embryo development of the selfed progeny. In the analysis of the offspring, we did not identify early embryonic arrest in *plt2 plt7* and *bbm plt7* double mutants (Table 1). In contrast, we were unable to recover a homozygous *plt2 bbm* mutant (as also described by Galinha et al., 2007 and Chen et al., 2022) and found that approximately 25% of *plt2/+ bbm* progeny seeds arrested development, becoming concave in shape (Fig. 3B; Table 1). To exclude the possibility that the observed phenotype is specific to *plt2* and *bbm* alleles generated by the T-DNA insertions in their coding sequences or the mixed ecotype background constituting the seedlings (Ws×Col-0), we additionally targeted both genes simultaneously with single-guide RNAs (sgRNAs) to generate loss-of-function alleles with CRISPR/Cas9 in Col-0 (Fig. 3A). Approximately a quarter of seeds in siliques of *plt2 bbm/+ -cr* were aborted, closely resembling the T-DNA mutant phenotype (Fig. 3B; Table 1). Moreover, we directly analyzed seed morphology in (T_1_) plants transformed with the CRISPR construct harboring homozygous *plt2* and *bbm* alleles in the inflorescences. In three independent plants*,* all seeds were aborted (Fig. 3B). These findings indicate that loss of both *PLT2* and *BBM* results in embryo lethality independent of the genetic background, consistent with observations of Chen et al. (2022) and Galinha et al. (2007). The frequency of arrested embryos was as expected for fully recessive alleles, indicating that the effect of the mutations occurs post-fertilization rather than being a maternal effect.

**Table 1.**
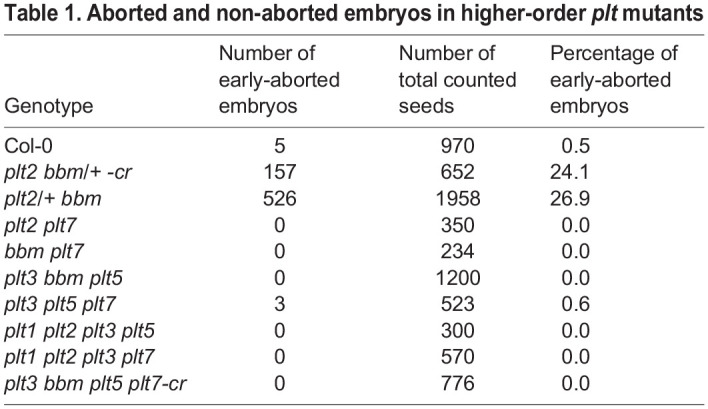
Aborted and non-aborted embryos in higher-order *plt* mutants

To confirm that the developmental defects occurred early in embryogenesis as reported by Chen et al. (2022), we examined the ovules of selfed *plt2/+ bbm* and *plt2 bbm/+ -cr* plants at the age at which globular embryos could be easily recognized within the ovule of wild-type plants. In *plt2/+ bbm* and *plt2 bbm/+ -cr* plants, however, embryos of the same age were inconspicuous in ∼25% of the ovules (Table 1). Microscopic examination of these ovules revealed that *plt2 bbm* embryos arrested development at very early stages in development. Of the observed *plt2 bbm* embryos, 69% (20/29) formed zygotes that could not divide at all and arrested immediately after fertilization, frequently becoming deformed in shape (Fig. 3D,F,G), whereas wild-type embryos were already at the globular stage (Fig. 3C,E). Although 24% (7/29) of *plt2 bbm* zygotes could progress to the one-cell stage, they often failed to correctly position the first asymmetric cell division (Fig. 3H), which was also observed in *plt2 bbm-cr* (Fig. 3I,J). Additional divisions were occasionally observed in the apical and basal lineages (7%; 2/29) and occurred atypically, i.e. at aberrant locations and in erratic planes, in both *plt2 bbm* (Fig. 3K,N) and *plt2 bbm-cr* (Fig. 3L). *PLT2* and *BBM* are thus required for specifying the first cell divisions after fertilization.

We next investigated whether other family PLT members are redundantly required during early embryo development by stacking other *plt* mutant alleles. We did not identify more frequent embryonic arrest in *plt3 bbm plt5* and *plt3 plt5 plt7-cr* triple mutants than in wild-type plants; nor was this observed in the quadruple mutants *plt1 plt2 plt3 plt5*, *plt1 plt2 plt3 plt7* and *plt3 bbm plt5 plt7-cr*, in which a *BBM* null allele was generated with CRISPR/Cas9 in the *plt3 plt5 plt7-cr* background (Table 1; Table S11). In addition, *plt3 bbm-2*, *plt1 plt3 bbm-2* and *plt1 plt2/+ plt3 bbm-2* seedlings are viable (Galinha et al., 2007). Taken together, these results demonstrate that PLT2 and BBM have a specific role in early development that is not shared by other PLT genes, likely through expression in the zygote.

### PLT gene expression in the *PLT2* or *BBM* expression domains rescues embryonic arrest

Given that PLT2 and BBM act in a redundant fashion, we examined whether PLT protein identity or spatiotemporal control of PLT gene expression was more important for progression of embryogenesis. We first explored whether *PLT2* and *BBM* are indeed interchangeable by introducing pPLT2::BBM-YFP and pBBM::PLT2-YFP promoter swap constructs in the *plt2/+ bbm* background, and by counting the number of wild-type and aborted embryos. Both swaps could almost fully rescue the *plt2/+ bbm* embryo phenotype (Table 2), indicating that *PLT2* and *BBM* expression functionally overlap, as expected. We then investigated whether *plt2/+ bbm* complementation with other PLT genes driven by *pPLT2* or *pBBM* could be established. We selected *PLT1* and *PLT3*, which showed a different expression domain than *PLT2* and *BBM*, and were minimally, if at all, expressed in the zygote and the apical cell of the one-cell-stage embryo (Fig. 3A,C), and subsequently introduced pPLT2::PLT1-YFP, pPLT2::PLT3-YFP and pBBM::PLT1-YFP in *plt2/+ bbm* mutants to determine the proportion of aborted ovules. Importantly, each of these promoter swap constructs could rescue the *plt2 bbm* phenotype (Table 2). We conclude that, despite the distinct expression patterns of PLT genes during early embryogenesis, the role of PLT2 and BBM in initiation of embryo development can be exerted by at least two other PLT gene family members normally not expressed at this stage. Therefore, regulation of PLT gene expression rather than protein-specific features is likely crucial during early embryogenesis.

**Table 2.**
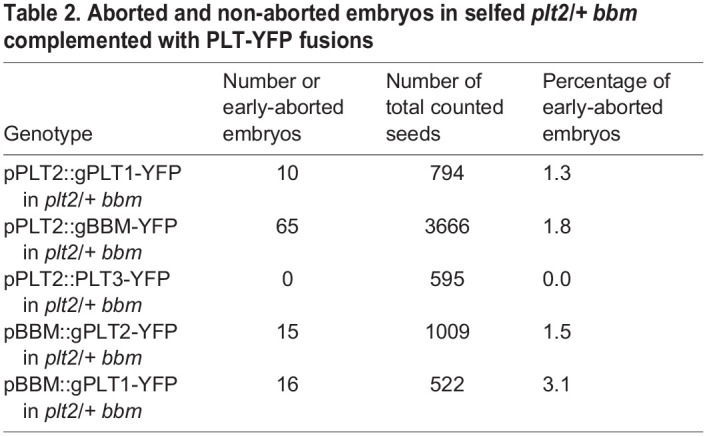
Aborted and non-aborted embryos in selfed *plt2/+ bbm* complemented with PLT-YFP fusions

## DISCUSSION

Here, we demonstrate that in zygotes and one-cell-stage embryos, a PLT-directed expression program is induced that resembles that of meristematic cells (Fig. 1). We show that in zygotes, only *PLT2* and *BBM* are transcribed, and that *PLT2*, *BBM* and *PLT7* are expressed in the apical and basal cells (Fig. 2). We reveal that PLT2 and BBM act redundantly to promote the transition of zygote to developing embryo. Absence of both PLT2 and BBM leads to embryonic arrest at the zygote or one-cell stage, a phenotype that could not be recapitulated with any other combination of *plt* single and higher-order mutants (Fig. 3; Table 1). Finally, we reveal with promoter swapping experiments that spatiotemporal control rather than PLT protein sequence identity is causal for the observed phenotype (Table 2).

By overlaying transcriptome datasets of the root meristematic zone (Li et al., 2016), shoot stem cells (Gutzat et al., 2020), and the quiescent center (Li et al., 2016), we identified a core set of 197 genes directly upregulated by PLTs (Fig. 1E; Table S2). Over 70% of this set was expressed in root stem(-like) cell lineages, zygotes, apical cells and basal cells from independent datasets (Fig. 1F). The genes in this set are diverse in nature and include many known factors in core developmental processes, some of which were expressed in all four zygotic/embryonic datasets (Tables S5-S8). This includes genes involved in regulation of chromatin [e.g. *DECREASED DNA METHYLATION 1* (Jeddeloh et al., 1999), and the two histone genes *HTB2* and *HTA8*], cell division and replication [e.g. *CYCLIN D5;1* (Wang et al., 2004), *PROLIFERA* (Springer et al., 1995) and *REPLICATION FACTOR C 2* (Shultz et al., 2007)], and the apical meristem patterning gene *SCARECROW* (Di Laurenzio et al., 1996; Bahafid et al., 2023). We also observed PLT core target genes that seemed to be expressed during specific developmental stages: for instance, the embryonic body axis initiator *MONOPTEROS* (Hardtke and Berleth, 1998) was only expressed in the apical and basal cells (Tables S6, S7) and expression of the shoot and embryo patterning gene *HANABA TARANU* (Zhao et al., 2004; Nawy et al., 2010) was only detected in the 24 HAP zygote apical and basal cells (Tables S5, S6, S8). We note, however, that the PLT core set also includes many genes with undescribed roles during (early) embryogenesis, or even of completely unknown function, which warrants further functional studies.

It has been thoroughly established that PLTs orchestrate a wide array of developmental processes (Horstman et al., 2014; Scheres and Krizek, 2018). Given that ectopic overexpression of all six PLT genes results in production of somatic embryos in *Arabidopsis* (Boutilier et al., 2002; Tsuwamoto et al., 2010; Horstman et al., 2017), it is not surprising that PLTs are also responsible for proper progression of zygotic embryogenesis. In line with the observations of Chen et al. (2022), we observed zygotic expression of *BBM*, but also of *PLT2*, albeit much more frequently (∼74% and ∼84%, respectively) than their reported ∼2%. This observation is supported by the 14 and 24 HAP zygote RNA-seq datasets (Zhao et al., 2019; Fig. S3A), in which expression levels similar to those observed in one-cell-stage embryos were detected. We thus presume this discrepancy is of a technical nature, possibly due to different construct expression levels, clearing/staining methodology, and/or the plant line used, and conclude that PLT2 and BBM are consistently present in zygotes. Importantly, research in other species corroborates the functional aspects of PLTs during early embryogenesis. For example, it has been shown in rice that ectopic overexpression of *OsBBM1* resulted in production of somatic embryos, and that seed viability of *osbbm1 osbbm2 osbbm3* triple mutants was severely compromised (Khanday et al., 2019). Understanding which PLTs guide early embryogenesis is vital in understanding how different transcriptional networks are employed to facilitate morphogenesis.

As we have shown through promoter swap assays (Table 2), it is not PLT protein sequence but rather expression pattern that reflects analogous gene function. Thus, predicting PLT function in other plant species based on orthologous relationships demands caution. In support of this notion, it has been shown that PLTs regulate a common set of target genes and that they all recognize an ANT-like consensus motif (Yano et al., 2009; Horstman et al., 2015; O'Malley et al., 2016; Santuari et al., 2016). Any PLT gene could at least partially rescue the root length and meristem size phenotypes observed in *plt1 plt2* mutants when driven by *pPLT2* (Galinha et al., 2007; Santuari et al., 2016), including *PLT7*. In addition, full complementation of the *plt3 plt5 plt7* lateral root phenotype was established when any PLT gene was expressed from the primordium-specific promoter *pPLT7-1.5 kb* (Du and Scheres, 2017). It is therefore likely that the embryonic defects in *plt2 bbm* mutants arise from the absence of PLTs during the zygote phase, leading to loss of meristematic potential, as *PLT2* and *BBM* appear to be the only expressed family members at this stage. In support of this notion, the majority of *plt2 bbm* embryos were not able to divide. Nevertheless, we cannot completely exclude the possibility that lack of strong PLT gene expression in the apical cell, where *PLT7* was only weakly expressed (Fig. 2G), in *plt2 bbm* underlies the observed embryonic arrest phenotypes in which cell divisions were able to take place (Fig. 3H-N).

From an evolutionary perspective, it is intriguing to observe redundancy of PLT2 and BBM, but not PLT1, during embryogenesis. PLT1 and PLT2 act redundantly to maintain the root apical meristem after germination in *Arabidopsis* (Aida et al., 2004; Galinha et al., 2007), but this redundancy is clearly not reflected in embryogenesis. *PLT1* and *PLT2* belong to the same gene clade within the *euANT* family, which exhibits little copy number variation as well as strongly conserved synteny across eudicots (Kerstens et al., 2020). How such similar paralogs developed differential spatiotemporal regulation in early embryos remains enigmatic, although differential expression was also observed in lateral root primordia (Du and Scheres, 2017). Identifying the relevant regulatory regions can shed light on the factors responsible for early embryonic gene expression of the PLT clade members.

In root meristems, PLT target gene analysis suggest that these factors promote stem cell maintenance, cell division and growth, and repress differentiation. The redundant and interchangeable roles of PLT genes in early embryogenesis lend support to the scenario that the initiation and maintenance of cell division and growth in embryos is mechanistically linked to meristematic programs. We presented evidence that direct PLT-regulated meristematic genes are already active during early embryogenesis, reinforcing this notion. It is therefore plausible that the evolution of the meristematic PLT growth control module required for initiation of embryonic divisions and meristematic potential predated and was adapted for embryonic growth in the context of seed development.

## MATERIALS AND METHODS

### DAP-seq

gDNA was extracted from 5 days post-germination Col-0 roots grown on ½ Murashige and Skoog (MS) medium including vitamins (Duchefa) and 3% plant agar (Duchefa). Briefly, tissue was pulverized with the TissueLyzer LT (QIAGEN) using two 30-s cycles at 30 Hz before extraction with 500 µl CTAB buffer (2% CTAB, 1.4 M NaCl, 20 mM EDTA, 100 mM Tris-HCl pH 8, 0.2% 2-mercaptoethanol) at 60°C for 30 min. The gDNA was extracted twice with 500 µl chloroform, then precipitated with 500 µl isopropanol. The pellet was washed with 1 ml 70% ethanol+10 mM ammonium acetate, resuspended in 250 µl TE buffer with 2.5 µg RNase A (Roche) and incubated for 30 min at 37°C. The RNA-free gDNA was precipitated with 250 µl isopropanol+25 µl 3 M sodium acetate, washed with 1 ml 70% ethanol, then finally resuspended in TE buffer. Then, 1 µg gDNA was fragmented in 400 ml TE buffer. Further library preparation steps were performed as described by Bartlett et al. (2017).

*In vitro* protein synthesis was performed with the TnT SP6 High-Yield Wheat Germ Protein Expression System (Promega) according to the manufacturer's instructions, using 5 µg plasmid as input. Further DAP steps were performed as described by Lai et al. (2020) for ‘single-protein DAP-seq’ with minor modifications. Briefly, 200 ng DAP-library was added to 48 µl TnT reaction, supplemented with 400 µl with DAP binding buffer (PBS, 0.005% NP40, 1 mM EDTA, pH 7.4) and incubated for 1 h at room temperature on a rotator, then 30 min on the tabletop. Next, 15 µl pre-washed anti-FLAG M2 magnetic beads (Sigma-Aldrich) were added to the library-TnT mix supplemented with DAP binding buffer to 1 ml and incubated at room temperature for 2 h on a rotator, and thereafter washed four times with 1 ml DAP binding buffer. The FLAG-protein-library complexes were eluted off the beads by twice incubating the beads with 400 µl elution buffer {380 µl PBS+20 µl 3xFLAG solution [5 mg/ml 3xFLAG peptide (APExBio), 0.125 M Tris-HCl, 0.25 M NaCl, pH 7.5]} on a rotator for 30 min at 4°C. The eluted 800 µl library was boiled for 10 min at 98°C, then column-purified with the GeneJET PCR Purification Kit (Thermo Fisher Scientific) using a 1:1:1 ratio of sample:GeneJET binding buffer:isopropanol. After 20 cycles of amplification, the libraries were size selected for fragments >200 bp and 150 bp-paired-end sequenced at GenomeScan BV on a NovaSeq6000 (Illumina) platform.

### ChIP-seq and DAP-seq analysis

Raw BBM ChIP-seq and all DAP-seq reads were obtained from Gene Expression Omnibus (accession numbers GSE52400 and GSE60143, respectively), as listed in Table S10. After adapter trimming with fastp v0.23.2 (Chen et al., 2018) reads were mapped to the TAIR10 genome with BWA-MEM v0.7.17 (Li, 2013 preprint). Secondary, unmapped, duplicate, and mapping quality <30 reads were removed with SAMtools v1.15 (Danecek et al., 2021) and Sambamba v0.6.6 (Tarasov et al., 2015). Peaks were called with MACS3 v3.0.0a7 (Zhang et al., 2008) at a *q*-value threshold of 0.05. To subtract background peak signal, HALO DAP (O'Malley et al., 2016), 3xFLAG-EGFP DAP (this study) and 35S::BBM/pBBM::GFP (Horstman et al., 2017) served as negative controls for the corresponding datasets associated with each study. Because PLT2 ChIP-seq reads (Santuari et al., 2016) were provided in color space format, they could not be analyzed directly with our pipeline; the called peaks from this study were adopted directly instead.

Nuclear peak overlap was determined by adding 150 bp flanking sequences to the called peaks, then reciprocally overlapping these sequence ranges using BEDtools (Quinlan and Hall, 2010). Because we obtained around tenfold more peaks from the two BBM ChIP-seq datasets than from the other sets with identical settings, overlap between these two datasets was omitted. Peaks scoring >75% reciprocal overlap were analyzed with MEME-ChIP (Machanick and Bailey, 2011) and annotated with HOMER (Heinz et al., 2010). Analysis steps were computationally parallelized with GNU Parallel (https://www.gnu.org/software/parallel/).

### PLT Regulome analyses

Regulome analyses were performed in R v4.0.3 (R Core Team, 2010), using packages dplyr v1.0.8 (https://github.com/tidyverse/dplyr, https://dplyr.tidyverse.org), ggplot2 v3.3.5 (Wickham, 2016), ggvenn v0.1.9 (https://CRAN.R-project.org/package=ggvenn), magrittr v2.0.1 (https://github.com/tidyverse/magrittr), readxl v1.4.3 (https://github.com/tidyverse/readxl), rstatix v0.7.0 (https://github.com/kassambara/rstatix), Seurat v5.0.1 (Satija et al., 2015), tibble v3.1.6 (https://github.com/tidyverse/tibble) and tidyr v1.1.4 (https://github.com/tidyverse/tidyr). PLT gene overexpression-responsive genes were obtained from (Santuari et al., 2016). RNA-seq data for the root meristem, shoot stem cells, 14 HAP/24 HAP zygotes, apical/basal cell and root cell lineages (sample: ‘col-0’) were retrieved from publicly available datasets (Li et al., 2016; Zhao et al., 2019; Gutzat et al., 2020; Zhou et al., 2020; Roszak et al., 2021; Shahan et al., 2022) Apart from the protophloem scRNA-seq, which was normalized previously in probabilistic manner, and the other single-cell lineages, which were converted to log_10_ (TP10k), each dataset was converted to log_10_ (TPM) values and averaged if more than one replicate was present. Genes were marked as being expressed if log_10_ (TPM) >0.75, based on density plots of this parameter. Shoot stem cell genes meeting this criterion were determined separately for 7-day-, 14-day- and 35-day-old plants, then concatenated. Genes in the phloem set were considered expressed if normalized counts >5, and genes in the other root lineages if log_10_(TP10k) >0.1. ‘Early’ (stem-like) and ‘late’ (differentiated) protophloem cells were defined as having pseudotime ≤1 and pseudotime ≥86, respectively, and genes not expressed in at least 10% of these ‘early’ or ‘late’ cells were removed. Likewise, stem(-like) and differentiated cells in the Shahan et al. (2022) dataset were defined as belonging to pseudotime groups ‘T0’/‘T1’ and ‘T8’/‘T9’, respectively, with the same 10% cut-off. Cumulative PLT gene expression was determined by summing the expression of all PLT genes. GO analysis was performed with agriGO v2.0 (Tian et al., 2017) against the TAIR10 background, using hypergeometric tests with Hochberg (FDR) correction. The GO network was made with Revigo (Supek et al., 2011) with default settings and visualized with Cytoscape v3.9.1 (Shannon et al., 2003).

### Plant material

*Arabidopsis thaliana* ecotypes Columbia-0 (Col-0) and Wassilewskija (Ws) were used. *plt1-4* and *plt2-2* alleles have been described by Aida et al. (2004) and *plt3-1* by Galinha et al. (2007). *bbm-1* (SALK_ 097021) was provided by the SALK Institute (Alonso et al., 2003). *plt5-2* and *plt7-1* have been described by Prasad et al. (2011). Higher-order T-DNA mutants were obtained by stacking above mutants. The *plt3 plt5 plt7-cr* triple mutant has been described by Kerstens et al. (2021). *plt3 bbm plt5 plt7-cr* was generated with CRISPR/Cas9 of *BBM* in *plt3 plt5 plt7-cr* (see ‘Cloning’ section). *plt2 bbm-cr* lines were created by simultaneously targeting *PLT2* and *BBM* sgRNAs (see ‘Cloning’ section); *plt2 bbm/+ -cr* was generated by selfing the backcross *plt2 bbm-cr #1×*Col-0. Growth conditions of plants were as described by Willemsen et al. (1998). Translational pPLT::PLT-YFP have been described by Aida et al. (2004), Galinha et al. (2007) and Prasad et al. (2011). Seed abortion was counted in maturing siliques (yellow) on multiple individuals per plant line.

### Cloning

The promoter swaps were generated in the Gateway vector pGreenII 227 using the promoters and genomic sequences of the translational fusions as described by Aida et al. (2004) and Galinha et al. (2007). *PLT2* and *BBM* CRISPR constructs were generated as described by Kerstens et al. (2021), except that we used a *Zea mays* codon-optimized intronized Cas9 protein (zCas9i; Grützner et al., 2021), using one sgRNA per gene. The zCas9i module pRPS5A::ΩTMV:zCas9i:NOSt was assembled by combining pICH41233-pRPS5A (Kerstens et al., 2021), pICH41402 (Addgene #50285; ΩTMV 5′UTR), pAGM47523 (Addgene #153221; zCas9i) and pICH41421 (Addgene #50339; NOSt) in pICH47742 (Addgene #48001) using Golden Gate cloning (Engler et al., 2014). All constructs were electroporated into *Agrobacterium tumefaciens* (C58C1.pMP90) and transformed into Col-0 wild-type plants as described by Clough and Bent (1998).

The *in vitro* translation vector pSPUTK-GG was designed by making the Gateway vector pSPUTK (Invitrogen) Golden Gate-compatible. pSPUTK amplicons were amplified with pSPUTK F1/R1 and pSPUTK F2/R2, and combined with a *lacZ* amplicon originating from pICH47751 (Addgene #48002) amplified with pSPUTK-*lacZ* F/R. pSPUTK-GG 3xFLAG-cPLT3 was made by first amplifying *cPLT3* (*AT5G10510.4*/*AT5G10510.6*) from Col-0 cDNA with BsaI-cPLT3 F and BsaI-cPLT3 R, then combining the amplicon with pICSL30005 (Addgene #50299; 3xFLAG) and pSPUTK-GG through Golden Gate cloning. pSPUTK-GG 3xFLAG-GFP was made in the same way using pICH41531 (Addgene #50321; EGFP) instead of an amplicon. Primers used to generate the constructs are listed in Table S12.

### Genotyping

The T-DNA insertions in *plt1-4*, *plt2-2*, *plt3-1*, *bbm-1*, *plt5*-*2* and *plt7-1* lines were verified by genotyping as described by Aida et al. (2004), Galinha et al. (2007) and Prasad et al. (2011). Mutant combinations were generated by crossing, and lines of different allelic combinations were selected from F2 populations by genotyping. *plt2* and *bbm* CRISPR target regions were amplified with *PLT2* CRISPR F/R and *BBM* CRISPR F/R, then Sanger sequenced with either primer (Table S12).

### Oligonucleotides

### Microscopy

For differential interference (DIC) microscopy, ovules were collected from developing siliques, cleared and mounted according to Willemsen et al. (1998). Confocal microscopy of PLT-YFP embryos was performed on dissected embryos mounted in 5% glucose. YFP was detected with an excitation wavelength of 488 nm. For visualization of *plt2 bbm* arrests, staining and clearing were performed on globular-stage ovules segregating the mutant allele using ClearSee and SCRI Renaissance 2200 as described by Tofanelli et al. (2019), and embryos were imaged using a 405 nm excitation laser. Zygotic PLT gene expression was analyzed by hand-pollinating stage 13 flowers, and subsequently fixing, clearing and staining 32 HAP whole siliques with ClearSeeAlpha and SCRI Renaissance 2200 as described by Kurihara et al. (2021), and confocal microscopy was performed as above.

## Supplementary Material



10.1242/develop.202527_sup1 Supplementary information

Table S1. Genes with a PLT binding peak within a [-4 kb, +4 kb] range from the TSS and direct PLT target genes upregulated during PLT overexpression.

Table S2. 197 overlapping direct PLT target genes in the RAM, SAM and QC.

Table S3. Gene ontology analysis of the 197 ‘core’ PLT regulome targets using hypergeometric tests with the Hochberg (FDR) correction.

Table S4. Biological process GO clustering analysis of the PLT ’core’ regulome.

Table S5. PLT ‘core’ targets expressed in the apical cell of the embryo (Zhou et al., 2020).

Table S6. PLT ‘core’ targets expressed in the basal cell of the embryo (Zhou et al., 2020).

Table S7. PLT ‘core’ targets expressed in 14 HAP zygotes (Zhao et al., 2019).

Table S8. PLT ‘core’ targets expressed in 24 HAP zygotes (Zhao et al., 2019).
