# Correction: PLETHORA transcription factors promote early embryo development through induction of meristematic potential

**DOI:** 10.1242/dev.204824

**Published:** 2025-04-11

**Authors:** Merijn Kerstens, Carla Galinha, Hugo Hofhuis, Michael Nodine, Renan Pardal, Ben Scheres, Viola Willemsen

There was an error in *Development* (2024) **151**, dev202527 (doi:10.1242/dev.202527).

Figure S1C contained an error. The corrected and original figures are shown below.

**Fig. S1 (corrected).**
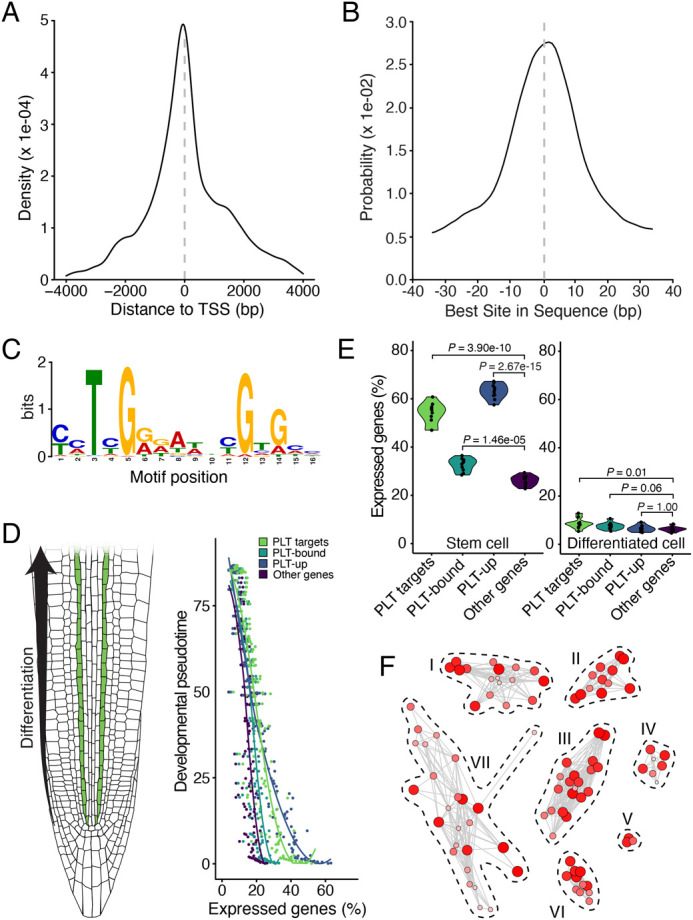


**Fig. S1 (original).**
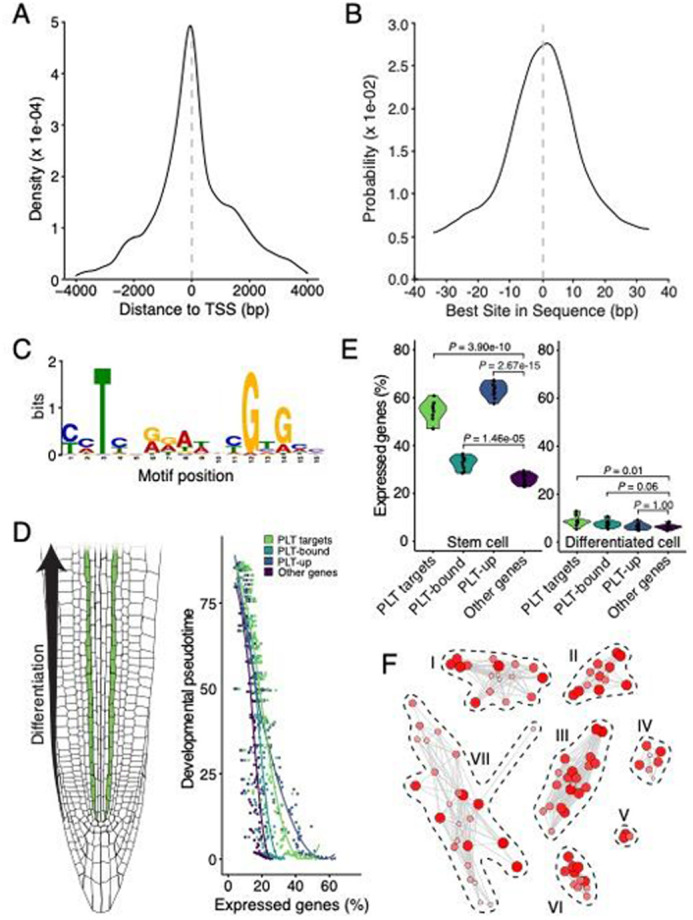


The online PDF of the supplementary file has been corrected.

The authors apologise for this error, which does not impact the results and conclusions of the paper.

