# Supplementary material for: PLETHORA transcription factors promote early embryo development through induction of meristematic potential
Source: Development. 2024 Jun 17;151(12):dev202527. doi: 10.1242/dev.202527 (PMC11234262; doi:10.1242/dev.202527)
Supplement: Supplementary information [file develop-151-202527-s1.pdf]

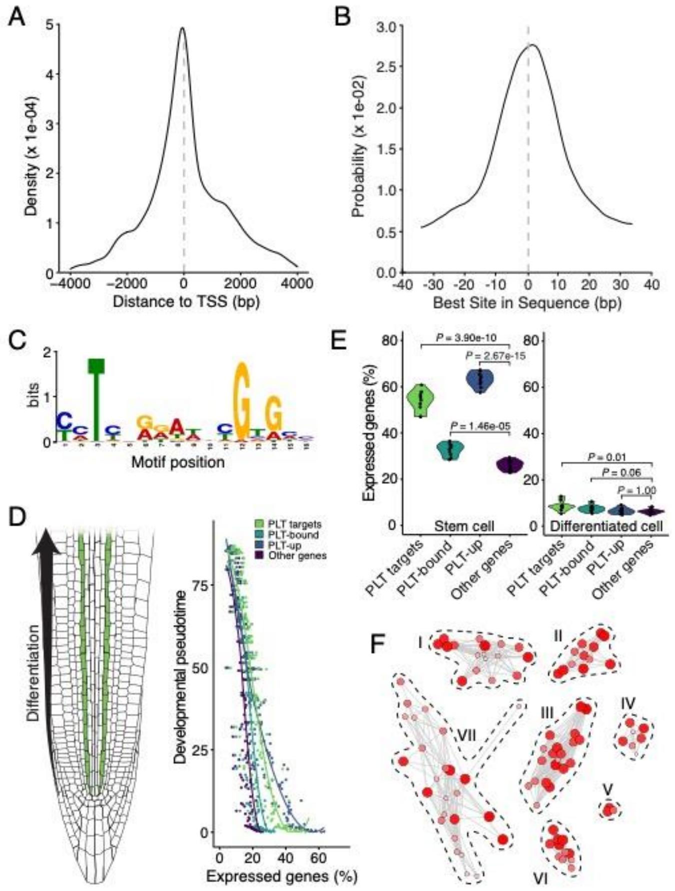

Fig. S1. PLTs bind to a canonical binding motif, and genes near these motifs are expressed primarily in meristematic cells. (A) Density plot of PLT ChIP/DAP-seq peaks around the TSS of target genes. (B) CentriMo output showing positions of PLT recognition sequence within PLT peaks, as depicted in the sequence log in (C). (D) Percentage of expressed PLT target (bound + upregulated), PLT-bound, PLT-upregulated and non-target genes over pseudotime, as depicted in the schematic root. The protophloem lineage is indicated. Each dot is the average value of cells in bins of 0.1 pseudotime (n > 1). Lines are loess regressions. (E) Percentage of expressed PLT target (bound + upregulated), PLT-bound, PLT-upregulated and non-target genes in stem cell-like and differentiated protophloem cells (one-tailed Welch's *t*-tests, Bonferroni correction, n = 10). (F) Network representation of the GO category 'biological process' for the 197 factors in the 'core' PLT regulome. Nodes are terms, edges depict relatedness between terms. Clusters are numbered and represent the following terms: regulation (I), response (II), development and morphogenesis (III), chromatin and cytoskeleton organization (IV), cell division (V), import, transport and localisation (VI), and (DNA) metabolism and modification (VII). Node size and colour scale with FDR values (i.e. bigger and redder is more significant).

Development • Supplementary information

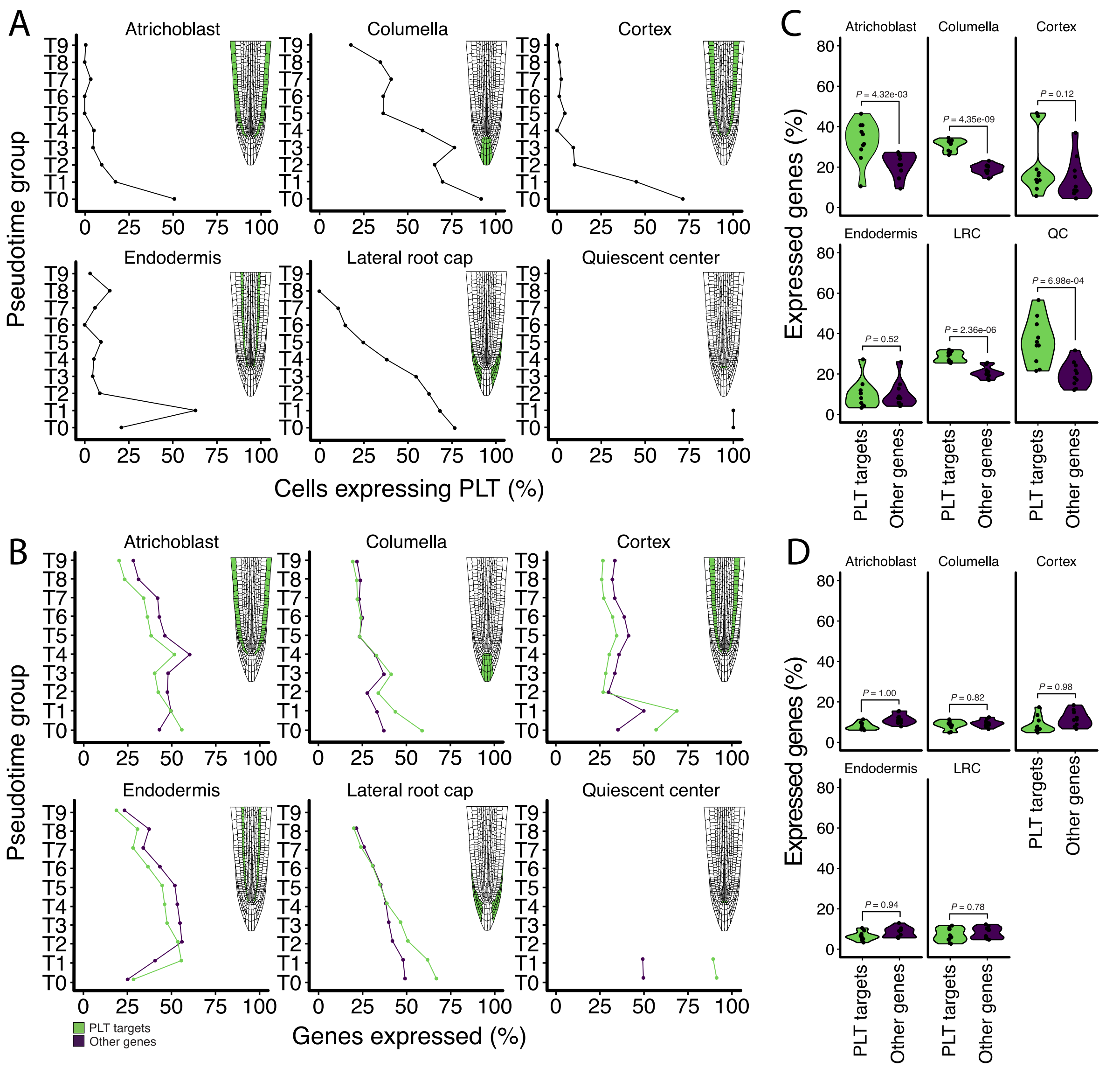

Fig. S2. *PLT* and upregulated direct PLT target gene expression is enriched in stem(-like) cells in various root cell lineages. (A) Percentage of atrichoblast, columella, cortex, endodoermis, lateral root cap and quiescent center cells expressing PLTs over ten developmental pseudotime groups. Time group 0 ('T0') contains the earliest, most stem-like cells, whereas T9 contains the latest, most differentiated cells. Dots are the average percentage within each time group (n ≥ 5 per group). Lineages are indicated on a schematic root axis. (B) Percentage of expressed PLT target and non-target genes over pseudotime groups in the cell lineages given in (A) (n ≥ 5 per group). (C,D) Percentage of expressed PLT target and non-target genes in the ten most stem cell-like (C) and most differentiated (D) cells for the indicated lineages. *P*-values are derived from one-tailed Welch's *t*-tests for all comparisons, except for the cortex (C,D) and the endodermis (C) comparisons, which rely on one-tailed Mann-Whitney U tests instead. LRC = lateral root cap, QC = quiescent center.

Development • Supplementary information

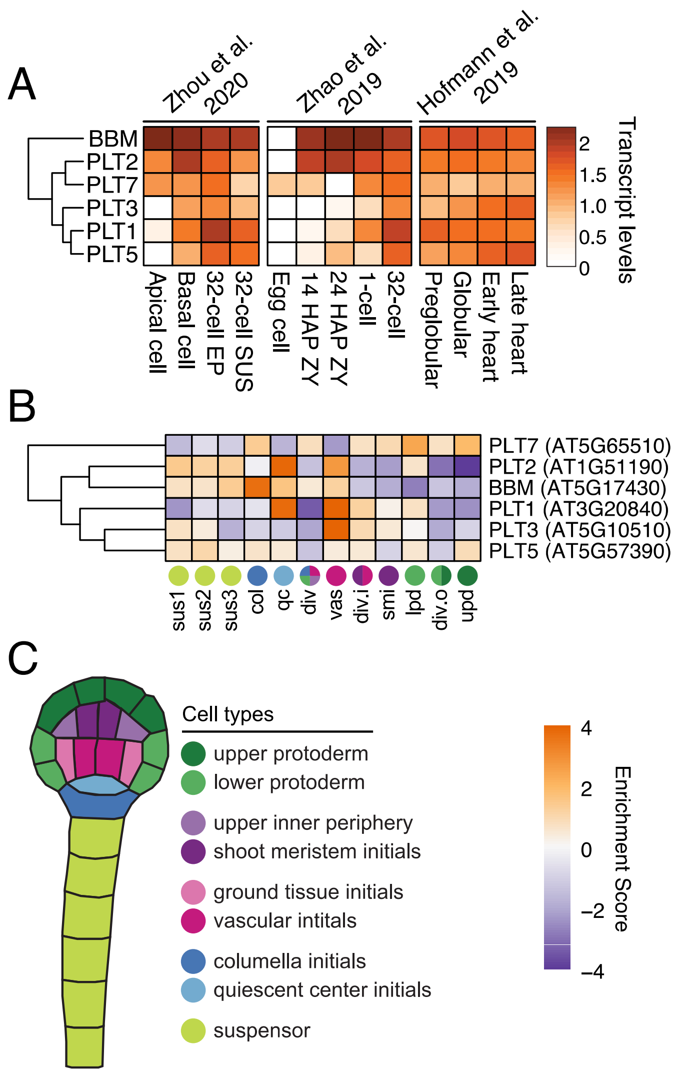

Fig. S3. *PLT* genes are expressed in partially overlapping expression domains in early and late embryogenesis. (A) *PLT* expression throughout embryogenesis (Hofmann et al., 2019; Zhao et al., 2019; Zhou et al., 2020). PLTs are clustered according to $\log_{10}$-transformed transcripts per million (TPM) values. (B) Single-cell *PLT* expression in globular embryo cell types as matrix and schematic heatmap (C). PLTs are clustered based on enrichment score (Kao et al., 2021).

Table S1. Genes with a PLT binding peak within a [-4 kb, +4 kb] range from the TSS and direct PLT target genes upregulated during PLT overexpression.

Available for download at
https://journals.biologists.com/dev/article-lookup/doi/10.1242/dev.202527#supplementary-data

Table S2. 197 overlapping direct PLT target genes in the RAM, SAM and QC.

Available for download at
https://journals.biologists.com/dev/article-lookup/doi/10.1242/dev.202527#supplementary-data

Development • Supplementary information

Table S3. Gene ontology analysis of the 197 'core' PLT regulome targets using hypergeometric tests with the Hochberg (FDR) correction.

Available for download at
https://journals.biologists.com/dev/article-lookup/doi/10.1242/dev.202527#supplementary-data

Table S4. Biological process GO clustering analysis of the PLT 'core' regulome.

Available for download at

https://journals.biologists.com/dev/article-lookup/doi/10.1242/dev.202527#supplementary-data

Table S5. PLT 'core' targets expressed in the apical cell of the embryo (Zhou et al., 2020).

Available for download at
https://journals.biologists.com/dev/article-lookup/doi/10.1242/dev.202527#supplementary-data

Table S6. PLT 'core' targets expressed in the basal cell of the embryo (Zhou et al., 2020).

Available for download at
https://journals.biologists.com/dev/article-lookup/doi/10.1242/dev.202527#supplementary-data

Table S7. PLT 'core' targets expressed in 14 HAP zygotes (Zhao et al., 2019).

Available for download at
https://journals.biologists.com/dev/article-lookup/doi/10.1242/dev.202527#supplementary-data

Table S8. PLT 'core' targets expressed in 24 HAP zygotes (Zhao et al., 2019).

Available for download at
https://journals.biologists.com/dev/article-lookup/doi/10.1242/dev.202527#supplementary-data

Table S9. ChIP/DAP-seq datasets used in this study.

Dataset	Tissue origin	Peaks	Overlap	--extsize	Reference
PLT1 DAP	Leaf	277	251	140	O'Malley et al. (2016)
PLT1 ampDAP	Leaf	137	137	117	O'Malley et al. (2016)
PLT3 DAP	Leaf	496	487	124	O'Malley et al. (2016)
PLT3 DAP	Root	1996	893	156	This study
PLT7 DAP	Leaf	655	617	175	O'Malley et al. (2016)
PLT7 ampDAP	Leaf	2251	1411	125	O'Malley et al. (2016)
PLT2 ChIP	Root	2482	1201	-	Santuari et al. (2016)
BBM ChIP	Seedling	20196	1617	260	Horstman et al. (2017)
BBM ChIP	Somatic embryo	16181	1355	265	Horstman et al. (2017)

Table S10. Peak overlap between *PLT* ChIP and DAP datasets. Numbers reported are shared peaks with at least one other dataset. Note that the *BBM* ChIP datasets were not directly compared with one another.

Dataset	Peaks shared
PLT1 DAP, leaf	269
PLT1 ampDAP, leaf	137
PLT3 DAP, leaf	505
PLT7 DAP, leaf	619
PLT7 ampDAP, leaf	1417
PLT3 DAP, root	907
PLT2 ChIP, root	1201
BBM ChIP, seedling	1617
BBM ChIP, somatic embryo	1355

Development • Supplementary information

Table S11. CRISPR mutants generated in this study. Mutation sites are indicated upstream of the PAM (N[GG] = 0) and downstream of the TSS (A[TG] = +1).

Gene	Genotype	Indel	bp from PAM	bp from TSS	Length (aa)
PLT2	Col-0	-	-	-	568
	plt2 bbm-cr T_1 #1	+ T	-4	+476	87
	plt2 bbm-cr T_1 #2	+ T	-4	+476	87
	plt2 bbm-cr T_1 #3	+ A	-5	+475	87
BBM	Col-0	-	-	-	584
	plt2 bbm-cr T_1 #1	+ T	-4	+21	15
	plt2 bbm-cr T_1 #2	+ A	-4	+21	15
	plt2 bbm-cr T_1 #3	+ A	-4	+21	15
	plt3 bbm plt5 plt7-cr	+ A	-4	+21	15

Table S12. Oligonucleotides

Oligo name	Sequence 5' - 3'
PLT2 sgRNA	CTTAGGAGTGAGCAAATCGG
BBM sgRNA	AACTCGATGAATAACTGGTT
PLT2 CRISPR F	GTTTGCAGCCATACTTGGAG
PLT2 CRISPR R	CTTTCACAGTGGCGACTTCT
BBM CRISPR F	GCCTCGGAAGAAATGAACAT
BBM CRISPR R	CACAAACCTCGGGAGTGACT
BsaI-cPLT3 F	AAAGGTCTCAAATGATGGCTCCGATGACG
BsaI-cPLT3 R	AAAGGTCTCAAAGCTTAGTAAGACTGATTAGGCCAGAGG
pSPUTK F1	ATAGAAGACATGCTTCCCTATAGTGAGTCGTATTAATTTCG
pSPUTK R1	ATAGAAGACATGTTCTCGCGGTATCATTGCAG
pSPUTK F2	ATAGAAGACATGAACCACGCTCACCGGCTCC
pSPUTK R2	ATAGAAGACATATGGTCTGCCAAAGTTGAGCGTT
pSPUTK-lacZ F	ATAGAAGACATCCATTGAGACCGCAGCTGGCAC
pSPUTK-lacZ R	ATAGAAGACATAAGCTGAGACCGTCACAGCTTGTCTGTAAG

Development • Supplementary information
