# Supplementary material for: PLETHORA transcription factors promote early embryo development through induction of meristematic potential
Source: Development. 2024 Jun 17;151(12):dev202527. doi: 10.1242/dev.202527 (PMC11234262; doi:10.1242/dev.202527)
Supplement: Supplementary file 1 [file develop-151-202527_review_history.pdf]

PLETHORA transcription factors promote early embryo development through induction of meristematic potential

Merijn Kerstens, Carla Galinha, Hugo Hofhuis, Michael Nodine, Renan Pardal, Ben Scheres and Viola Willemsen
DOI: 10.1242/dev.202527

Editor: Dominique Bergmann

Review timeline

Original submission:	10 November 2023
Editorial decision:	3 January 2024
First revision received:	1 May 2024
Accepted:	10 May 2024

Original submission

First decision letter

MS ID#: DEVELOP/2023/202527

MS TITLE: PLETHORA transcription factors promote early embryo development through induction of meristematic potential

AUTHORS: Merijn Kerstens, Carla Galinha, Hugo Hofhuis, Michael Nodine, Renan Pardal, Ben Scheres, and Viola Willemsen

I have now received all the referees' reports on the above manuscript, and have reached a decision. The referees' comments are appended below, or you can access them online: please go to BenchPress and click on the 'Manuscripts with Decisions' queue in the Author Area.

As you will see, the referees express considerable interest in your work, but have some significant criticisms and recommend a substantial revision of your manuscript before we can consider publication. If you are able to revise the manuscript along the lines suggested, which may involve further experiments, I will be happy receive a revised version of the manuscript. Both reviewers, but especially reviewer one, give many useful suggestions to improve the manuscript and I agree with the request to analyse additional datasets. For this revision, I do not think it is necessary to create new mutants or transgenics, but rather in those cases suggested by reviewer to modify the claims in the text. There are strengths and differences in this work vs the PNAS paper that came out recently and it would be good to simply highlight your important contributions and focus--the way the PNAS paper is currently described and cited could be smoother. I also agree with Reviewer 1 that the statement in line 193 absolutely needs to be revised.

Your revised paper may be re-reviewed by one or more of the original referees, and acceptance of your manuscript will depend on your addressing satisfactorily the reviewers' major concerns. Please also note that Development will normally permit only one round of major revision. If it would be helpful, you are welcome to contact us to discuss your revision in greater detail. Please send us a point-by-point response indicating your plans for addressing the referees' comments, and we will look over this and provide further guidance.

Please attend to all of the reviewers' comments and ensure that you clearly highlight all changes made in the revised manuscript. Please avoid using 'Tracked changes' in Word files as these are lost

© 2024. Published by The Company of Biologists under the terms of the Creative Commons Attribution License (https://creativecommons.org/licenses/by/4.0/).

in PDF conversion. It is essential to also provide a point-by-point response detailing how you have dealt with the points raised by the reviewers in the 'Response to Reviewers' box. If you do not agree with any of their criticisms or suggestions please explain clearly why this is so.

Reviewer 1

Advance summary and potential significance to field

Kerstens et al explore the role of PLT transcription factors during early embryogenesis. While PLTs are known to regulate meristematic divisions in root and shoot meristems, their role during embryogenesis was unknown. Since PLTs are linked with meristematic activity they aim to determine whether PLTs are required for a similar program during embryogenesis. They complement existing PLT ChIP and DAPseq experiments with a root PLT3 DAPseq dataset and use that and existing datasets to identify genes that are bound and upregulated by PLTs and that are found across apical meristems expression datasets. They find that more than 75% of these PLT targets are expressed in both the apical and basal cell of the one cell embryo. Next they check which of the PLTs are expressed at this stage and find that PLT2, PLT4 (BBM) and PLT7 are expressed in both cells which some others are only expressed in the basal cell. Plt2 bbm is known to be embryo lethal and the authors find with both existing TDNA and new CRISPR alleles that most plt bbm arrest at zygote stage and those that progress arrest before globular stage. This double mutant can be complemented by PLT1 or PLT3 when expressed under the PLT2 or PLT4 showing that expression domain rather than protein sequence plays a key role in diversification. It is a nice story that shines a new light on the control of meristematic potential during early embryogenesis.

Comments for the author

Major
- The main argument for novelty and being separate from the Chen 2022 PNAS story is the focus on generic (apical) meristematic potential controlled by the PLTs. I would encourage the authors to then place more emphasis on this in the results. 184 PLT core target genes are a useful tool for finding commonalities across cells but I would be interested in seeing more on them and/or seeing more context. How many genes across these tissues that are not bound/regulated by PLTs? If more than 75% of PLT core targets is also present in the apical and basal cell, is that meaningful? Is it more than the non-PLT targets that are expressed across apical meristems? Or if it is not numbers that matter, then are there genes with particular meristematic potential in these 184 that deserve highlighting? Right now, we only get 5 lines (179-184) focused on what's in this list. I suggest expanding this to match with the described focus.
- Why is a phloem specific dataset being used to limit the PLT target list and/or to describe their association with stemness? I agree that including a single cell dataset adds more resolution but then why not take one that includes all root meristem cells such as (Denyer 2019, Shahan 2022, Wendrich 2020) and/or the shoot apex (Zhang 2021). I suggest including a non cell type specific single cell dataset to make sure no phloem specific effects snuck in.
- When describing the early embryonic phenotype the description of the different classes of phenotypes relies on the observation of 29 embryos. This is very little and not enough to make the claims in lines 266-268 that are based on 2 embryos. I'd advise looking at additional embryos (80-100) or not making claims such as 'rarely'. Finally, when looking at figure 3, there are already 3 such embryos, in panels K, M and N. These are all said to be plt2 bbm embryos with this division phenotype, are these from separate experiments? I suggest either looking at additional embryos or adjusting the claims.
- With the majority of plt2 bbm embryos arrested at zygote stage it would make sense to show expression of PLT2 and BBM at this stage. Chen 2022 reports very low expression for BBM in the zygote and it would be useful to see whether PLT2 and BBM are expressed according to your reporter lines. In addition, this zygote arrest means that I overall think it would be more interesting to focus on the zygote instead of the apical/basal cell expression of PLT targets. I suggest adding zygote pictures and describing zygote expression of PLT core genes.

Minor
- Is expression in the basal cell important at all? The essential ones here (PLT2 and BBM) seem to be unique in their apical cell expression (and potentially zygote). I would have loved to see

© 2024. Published by The Company of Biologists under the terms of the Creative Commons Attribution License (https://creativecommons.org/licenses/by/4.0/).

if the plt2 bbm phenotype could be complemented with just zygote+apical or zygote+basal expression. My hypothesis would be that zygote+apical might be sufficient.

- In line 122-124 you summarize all the results of the Chen 2022 paper except for the embryo phenotype and BBM expression pattern. I realize that with the timing your plt2 bbm embryonic phenotype was available earlier but describing all but the most relevant finding from that story here feels inappropriate.
- In line 142-143 I think it's better to refer to apical meristems since no data from lateral or intercalary meristems were used. Same in line 179.
- Just out of curiosity: why did you only continue working with genes upregulated by PLTs? Line 152. Why exclude repressed targets? I get that for Figure 1B-D this is more relevant but overall it might be good to considering genes repressed by PLTs as well.
- Line 179: Figure 1E instead of C.
- Line 185. It's unclear why the apical and basal cell of the one cell embryo are the focus of this story instead of for instance only the apical cell or the zygote. It feels a bit out of nowhere the way it's written now. And later it seems that the zygote would have been a better choice.
- Line 193. This statement makes no sense to me. Where does the journey start in the author's opinion? At the zygote? Where is the line drawn between stem cell and 'in transit'? What is a good measure of stemness? I much prefer the view that stemness is a trait that is generally present as a gradient.

Please consider rephrasing this.

- Line 283-286 is confusing to me. This seems like the obvious part. I would leave this out and focus on the complementation with the other PLTs, that is the interesting part in my opinion.
- With the focus on meristematic potential during embryogenesis. In the discussion there could again be more elaboration on this PLT core set of genes.
- Line 299. Instead of PLT response I'd consider saying that at this stage PLT is inducing programs (or genes) similar to those it induces in meristematic cells.
- Line 303. Shortly after fertilization is very relative. The zygote does elongate which to me means it arrests shortly before division instead of shortly after fertilization.
- Line 311-312. I'm not sure how being able to promote somatic embryogenesis links to the zygote? As far as I know the first steps of somatic embryogenesis are not stereotypical in the same way and other inducers of somatic embryogenesis such as the LAFLs are not involved in zygote development.

Consider rephrasing.

- Line 319-329 would make more sense to go after the following paragraph.

Now the paragraph after it feels like it negates it.

- Line 339-344. Checking PLT7 expression in the zygote quickly addresses this. In addition, theoretically do you think PLT2::PLT7 could rescue the plt2 bbm mutant?
- Line 351-353. Could consider saying 'required for initiation embryonic divisions and meristematic potential'.

<u>Reviewer 2</u>

Advance summary and potential significance to field

The manuscript entitled "PLETHORA transcription factors promote early embryo development through induction of meristematic potential" by Kerstens et al. reports the role of PLTs in early embryo development. The authors show that PLT targets are related to meristem and embryo development, then they observe the expression patterns of PLT2/BBM and other PLTs during embryo development, and finally they show that the plt2 bbm double mutant exhibits sever defects in embryo development. Overall, this manuscript provides key information on the role of PLTs in embryos.

Comments for the author

I have several suggestions for the authors:

1) The authors indicate the role of PLT2/BBM in early embryo development.
WOX2/WOX9 are also key genes in the early stage of embryo development and are involved in specification of apical/basal cell fates. What are the expression patterns or levels of WOX2/WOX9

© 2024. Published by The Company of Biologists under the terms of the Creative Commons Attribution License (https://creativecommons.org/licenses/by/4.0/).

in the plt2 bbm double mutant embryos? Or What are the patterns or levels of PLT2/BBM in the wox9/wox2 backgrounds? It will be very important to show the regulations of PLTs and WOXs. Or alternatively, the authors should at least have a very comprehensive discussion on the relations between PLTs and WOXs, and provide some possibilities/potential roles of PLTs and WOXs in early embryo development.

2) The expression patterns of PLTs in late embryo development are also very interesting. Is it possible to move some of the data in Figure S2 to the main text?

3) Detailed information of PLT and BBM CRISPR/Cas9 mutants might be added into Supplemental Figures.

First revision

Author response to reviewers' comments

Reviewer 1 - major comments
-The main argument for novelty and being separate from the Chen 2022 PNAS story is the focus on generic (apical) meristematic potential controlled by the PLTs. I would encourage the authors to then place more emphasis on this in the results. 184 PLT core target genes are a useful tool for finding commonalities across cells but I would be interested in seeing more on them and/or seeing more context. How many genes across these tissues that are not bound/regulated by PLTs? If more than 75% of PLT core targets is also present in the apical and basal cell, is that meaningful? Is it more than the non-PLT targets that are expressed across apical meristems? Or if it is not numbers that matter, then are there genes with particular meristematic potential in these 184 that deserve highlighting? Right now, we only get 5 lines (179-184) focused on what's in this list. I suggest expanding this to match with the described focus.

Response: We thank reviewer 1 for this good suggestion and agree that more context is helpful to place the percentages of Fig. 1F into perspective. For this reason, we have adapted the analysis to compare the percentage of expressed PLT core targets to the percentage of expressed non-PLT core targets. We have also calculated the log_2 enrichment of PLT core target expression over non-PLT core target expression. Moreover, we incorporated more tissue lineages from Shahan et al., 2022 (scRNA-seq of roots; more on that below) and Zhao et al., 2019 (zygote data; more on that below), and added extra supplemental data for the PLT core targets overlapping with the 14 HAP zygote (Data S5), 24 HAP zygote (Data S6), apical cell (Data S7) and basal cell (Data S8). From this analysis, it becomes clear that all stem(-like) cells in the various root lineages are enriched in the percentage of expressed PLT core target genes, and that the zygote, apical and basal cell follow this trend. In contrast to our previous analysis, this even more strongly corroborates the idea that the PLT regulome in stem(-like) and early embryonic cells is shared (see lines 177-193 and 209-221). Methods: lines 475-489.

In terms of the genes within the PLT cores set, we now cautiously describe specific genes from the core set in our manuscript, because we do not want to cherry-pick 'interesting' candidates too much. We have included a small section in the discussion to this point (see lines 345-363). For the overall picture, we think it is better to focus on the general functions of the PLT core targets, which we address with GO network analysis (see Fig. S1F, Data S3, Data S4 and see lines 201-208).

-Why is a phloem specific dataset being used to limit the PLT target list and/or to describe their association with stemness? I agree that including a single cell dataset adds more resolution but then why not take one that includes all root meristem cells such as (Denyer 2019, Shahan 2022, Wendrich 2020) and/or the shoot apex (Zhang 2021). I suggest including a non cell type specific single cell dataset to make sure no phloem specific effects snuck in.

Response: It is true that filtering the PLT core targets with a phloem dataset could introduce a bias. We therefore changed our approach: we first define the PLT core targets as PLT target (bound + upregulated) genes that are expressed in SAM stem cells (Gutzat et al., 2020), root meristematic zone (Li et al., 2016), and the quiescent center (Li et al., 2016). Thus, we omitted the phloem set from this step. This yielded a list of 197 (previously 184) PLT core target genes. We then checked the percentage of these genes that is expressed in different scRNA-seq root tissues -

© 2024. Published by The Company of Biologists under the terms of the Creative Commons Attribution License (https://creativecommons.org/licenses/by/4.0/).

we specifically used root cell lineages, as PLTs are present in a gradient there (unlike in the shoot apex) so we could check the expression of PLT targets over this gradient (which would correlate with developmental pseudotime). This includes the phloem lineage (Roszak et al., 2021) and various lineages from the Shahan et al. (2022) root atlas. For this non-cell type specific data resource, we first investigated how the *PLT* genes are expressed in the different developmental pseudotime groups (as defined by Shahan et al.). In the atrichoblast, columella, cortex, endodermis and lateral root cap, we observed a *PLT* expression gradient over pseudotime as observed in reporter lines of previous studies (Fig. S2A), so we used these lineages for further analysis. We also included QC cells as control, which all expressed *PLTs* (Fig. S2A). We then checked whether we also observed a gradient-wise expression of the 334 PLT target genes in these lineages. We did not clearly see the gradient that we saw in the protophloem lineage, likely because the Shahan data use a different pseudotime metric than in the Roszak data, but we did see more expression of PLT target genes over non-target genes in the earliest pseudotime groups (except for the endodermis) (Fig. S2B), which we could statistically support for the atrichoblast, columella, lateral root cap, and quiescent center (Fig. S2C). Note that we previously used only five cells to test for significant differences between the stem-like and the differentiated cells (in the Roszak data), but we changed this number to ten cells to account for the greater variation in the Shahan datasets. We then used the early stem(-like) pseudotime groups ('T0' and 'T1') and differentiated, late pseudotime groups ('T8' and 'T9') to address how the 197 PLT core targets were expressed (Fig. 1F). As described in our response above, we see enrichment in the expression of PLT core target genes over non-PLT core target genes in every cell type from the Shahan dataset. Therefore, we now show that this observation is not limited to the phloem lineage. See lines 177-193.

-When describing the early embryonic phenotype the description of the different classes of phenotypes relies on the observation of 29 embryos. This is very little and not enough to make the claims in lines 266-268 that are based on 2 embryos. I'd advise looking at additional embryos (80-100) or not making claims such as 'rarely'. Finally, when looking at figure 3, there are already 3 such embryos, in panels K, M and N. These are all said to be plt2 bbm embryos with this division phenotype, are these from separate experiments? I suggest either looking at additional embryos or adjusting the claims.

Response: We thank the reviewer for pointing this out. Although we only report 29 embryos here, these count data originate from a single experiment, which we have tried to clear up (lines 297, 301, 303-304). Also, we actually analyzed about ten-fold that number - the great majority of these embryos do not have a clear remnant by the time we analyze, because the arrested zygotes seem to have disintegrated. This is also reported in Chen et al., 2022 (Fig. 2F, they show ~90-95% 'empty' ovules). Nevertheless, we agree that it might be inaccurate to call an event 'rarely' if it occurs in 2/29 embryos. We have therefore adjusted the claim in the text (line 302). Regarding there being three division defect embryos in panels K, M and N, reviewer 1 is correct that this seems to contradict our '2/29' statement. However, as we pointed out, the '29 embryos'-count data originates from a single experiment in which we addressed the frequency of the different mutations in the *plt2/+ bbm* line; the images in K, M, and N do not necessarily stem from this experiment. We have tried to clear this up in the main text as mentioned and have now indicated in Fig. 3 that panels I, J and L are *plt2 bbm-cr* mutants.

-With the majority of plt2 bbm embryos arrested at zygote stage it would make sense to show expression of PLT2 and BBM at this stage. Chen 2022 reports very low expression for BBM in the zygote and it would be useful to see whether PLT2 and BBM are expressed according to your reporter lines. In addition, this zygote arrest means that I overall think it would be more interesting to focus on the zygote instead of the apical/basal cell expression of PLT targets. I suggest adding zygote pictures and describing zygote expression of PLT core genes.

Response: Reviewer 1 makes an important point here that we overlooked. We have performed the experiments as suggested and analyzed expression of the PLT marker lines in the elongated zygote and found that *PLT2* and *BBM* are expressed in elongated zygotes, but not any of the other *PLT*s (Fig. 2 and lines 228-229, 253-256, 271-272, 337-338, 369; methods in lines 545-551). As alluded to by reviewer 1, this means that it became very interesting for us to focus more on the zygote, in addition to the apical and basal cell. We have made adjustments to our manuscript in various locations to create this focus (lines 81, 133-134, 142, 144, 146, 217, 224, 228, 247, 266, 312-313, 336-337, 341, 376, 413), and, as mentioned before, we have added the zygote expression of PLT core targets versus non-PLT targets to Fig. 1F.

© 2024. Published by The Company of Biologists under the terms of the Creative Commons Attribution License (https://creativecommons.org/licenses/by/4.0/).

Reviewer 1 - minor comments
-Is expression in the basal cell important at all? The essential ones here (PLT2 and BBM) seem to be unique in their apical cell expression (and potentially zygote). I would have loved to see if the plt2 bbm phenotype could be complemented with just zygote+apical or zygote+basal expression. My hypothesis would be that zygote+apical might be sufficient.

Response: We think reviewer 1's hypothesis may be correct, or, alternatively, zygote expression alone vould be enough to complement the *plt2 bbm* arrest phenotype. For us, this would be a point that could be addressed in a follow-up manuscript. We now briefly mention this in the discussion (lines 390-397).

-In line 122-124 you summarize all the results of the Chen 2022 paper except for the embryo phenotype and BBM expression pattern. I realize that with the timing your plt2 bbm embryonic phenotype was available earlier but describing all but the most relevant finding from that story here feels inappropriate.

Response: We now mention the *BBM* expression pattern and arrest phenotype in the introduction and discussion (lines 121-127 and 368-370). We also refer to them in the results (lines 288, 291-294).

-In line 142-143 I think it's better to refer to apical meristems since no data from lateral or intercalary meristems were used. Same in line 179.

Response: We have revised these lines as suggested.

-Just out of curiosity: why did you only continue working with genes upregulated by PLTs? Line 152. Why exclude repressed targets? I get that for Figure 1B-D this is more relevant but overall it might be good to considering genes repressed by PLTs as well.

Response: It would certainly be interesting to consider repressed targets as well. However, since our analysis is based on whether genes are expressed across various tissues or not, we would not be able to identify whether genes are directly repressed by PLTs, or whether they were not expressed to begin with due to other reasons (for example, due to chromatin state). We thus are of opinion that our analysis cannot be used to draw conclusions about repressed target genes, hence we did not include it in our manuscript.

-Line 179: Figure 1E instead of C.

Response: We have revised this line as suggested.

-Line 185. It's unclear why the apical and basal cell of the one cell embryo are the focus of this story instead of for instance only the apical cell, or the zygote. It feels a bit out of nowhere the way it's written now. And later it seems that the zygote would have been a better choice.

Response: Indeed, we have now shifted the focus of the manuscript to 'early embryonic' cells, including the zygote (e.g. lines 81, 133-134, 142, 144, 146, 217, 224, 228, 247, 266, 312-313, 336-337, 341, 376, 413). We introduce the overall research question in the first sentence of our 'Results' section (lines 143-144).

-Line 193. This statement makes no sense to me. Where does the journey start in the author's opinion? At the zygote? Where is the line drawn between stem cell and 'in transit'? What is a good measure of stemness? I much prefer the view that stemness is a trait that is generally present as a gradient.
Please consider rephrasing this.

Response: We agree that this sentence is too vague and subjective. We have rephrased this line and merged it with the one in front.

-Line 283-286 is confusing to me. This seems like the obvious part. I would leave this out and focus on the complementation with the other PLTs, that is the interesting part in my opinion.

Response: Although it is indeed most interesting that other PLTs (i.e. the ones not expressed in the zygote/apical cell) can rescue the *plt2 bbm* phentoype, we do not agree that it is 'obvious' that pPLT2::BBM-YFP and pBBM::PLT2-YFP would rescue the *plt2 bbm* and that this experiment should be left out. These two constructs are important controls highlighting that PLT2 and BBM can rescue the *plt2 bbm* mutant, and that they are interchangeable. In our opinion, this sets up the reasoning for checking whether other PLTs can also rescue the *plt2 bbm* phenotype.

© 2024. Published by The Company of Biologists under the terms of the Creative Commons Attribution License (https://creativecommons.org/licenses/by/4.0/).

-With the focus on meristematic potential during embryogenesis. In the discussion there could again be more elaboration on this PLT core set of genes.

Response: As we mentioned before, this would, in our opinion, entail cherry-picking the most 'interesting' genes from our PLT core target set, so we have only dedicated a small section in the discussion to this point (see lines 345-363).

-Line 299. Instead of PLT response I'd consider saying that at this stage PLT is inducing programs (or genes) similar to those it induces in meristematic cells.

Response: We have rephrased this first sentence of the discussion.

-Line 303. Shortly after fertilization is very relative. The zygote does elongate which to me means it arrests shortly before division instead of shortly after fertilization.

Response: We agree that this line was too relative and rephrased this line as suggested.

-Line 311-312. I'm not sure how being able to promote somatic embryogenesis links to the zygote? As far as I know the first steps of somatic embryogenesis are not stereotypical in the same way and other inducers of somatic embryogenesis such as the LAFLs are not involved in zygote development. Consider rephrasing.

Response: Until now there are no reports on the link between zygotic expression and somatic embryogenesis, but we (and others) have shown that overexpression of BBM, through activation of *LAFL* genes (Horstman et al., 2017), results in somatic embryogenesis, which might indicate that BBM is the key factor in these two processes to activate embryogenesis programs.

-Line 319-329 would make more sense to go after the following paragraph. Now the paragraph after it feels like it negates it.

Response: We have switched these paragraphs as suggested.

-Line 339-344. Checking PLT7 expression in the zygote quickly addresses this. In addition, theoretically do you think PLT2::PLT7 could rescue the plt2 bbm mutant?

Response: Indeed, PLT7 was not expressed in the zygote (Fig 2F), showing that reviewer 1's suspicions that zygotic PLT expression is important was correct. And yes, since PLT7 has been shown to be able to rescue the *plt1 plt2* root phenotype (Santuari et al., 2016) and has a similar binding site in vitro, we suspect that it could rescue the *plt2 bbm* mutant.

-Line 351-353. Could consider saying 'required for initiation embryonic divisions and meristematic potential'.

Response: We have integrated the suggestion in these lines.

Reviewer 2 - comments

-The authors indicate the role of PLT2/BBM in early embryo development. WOX2/WOX9 are also key genes in the early stage of embryo development and are involved in specification of apical/basal cell fates. What are the expression patterns or levels of WOX2/WOX9 in the plt2 bbm double mutant embryos? Or What are the patterns or levels of PLT2/BBM in the wox9/wox2 backgrounds? It will be very important to show the regulations of PLTs and WOXs. Or alternatively, the authors should at least have a very comprehensive discussion on the relations between PLTs and WOXs, and provide some possibilities/potential roles of PLTs and WOXs in early embryo development.

Response: We thank reviewer 2 for highlighting the role of *WOX2* and *WOX9* in early embryogenesis, and we certainly recognize the importance of these two genes. However, we think that experiments or a discussion on the roles of *WOX* genes is beyond the scope of our manuscript. We aim here to highlight the roles of PLT target genes, not WOX target genes, and neither WOX2 nor WOX9 are upregulated PLT-bound targets (only WOX9 is bound by PLTs). We do concur with the reviewer that this would be an interesting topic for a follow up study.

-The expression patterns of PLTs in late embryo development are also very interesting. Is it possible to move some of the data in Figure S2 to the main text?

Response: Yes, we agree that the expression patterns during late embryogenesis in Fig. S2 would also suit the main text. We have adapted Fig. 2 accordingly to focus on the expression patterns and have moved the RNA-seq heatmaps to Fig. S3. We have also placed more emphasis on these results in lines 243-246.

© 2024. Published by The Company of Biologists under the terms of the Creative Commons Attribution License (https://creativecommons.org/licenses/by/4.0/).

-Detailed information of PLT and BBM CRISPR/Cas9 mutants might be added into Supplemental Figures.

Response: Indeed, it is important that it is clear what the exact nature of the various CRISPR mutations is, and we already show this information in the supplemental tables (Table S3). We do not think it is necessary to add more information in the supplemental figures about the mutations.

Other changes

-Fig. 1A/S1E: We have changed the schematic diagram to more accurately represent an Arabidopsis root.

-Fig. 3A: We have removed the gene model for *PLT7*, since we do not show any embryonic phenotypes in this panel that include mutations in this gene.

-Figure legends have been adapted to suit the edited figures.

Second decision letter

MS ID#: DEVELOP/2023/202527

MS TITLE: PLETHORA transcription factors promote early embryo development through induction of meristematic potential

AUTHORS: Merijn Kerstens, Carla Galinha, Hugo Hofhuis, Michael Nodine, Renan Pardal, Ben Scheres, and Viola Willemsen

I am happy to tell you that your manuscript has been accepted for publication in Development, pending our standard ethics checks.

Reviewer 1

Advance summary and potential significance to field

Kerstens et al explore the role of PLT transcription factors during early embryogenesis. While PLTs are known to regulate meristematic divisions in root and shoot meristems, their role during embryogenesis was unknown. Since PLTs are linked with meristematic activity they aim to determine whether PLTs are required for a similar program during embryogenesis. They complement existing PLT ChIP and DAPseq experiments with a root PLT3 DAPseq dataset and use that and existing datasets to identify genes that are bound and upregulated by PLTs and that are found across apical meristems expression datasets. They find that more than 75% of these PLT targets are expressed in both the apical and basal cell of the one cell embryo. Next they check which of the PLTs are expressed at this stage and find that PLT2, PLT4 (BBM) and PLT7 are expressed in both cells which some others are only expressed in the basal cell. Plt2 bbm is known to be embryo lethal and the authors find with both existing TDNA and new CRISPR alleles that most plt bbm arrest at zygote stage and those that progress arrest before globular stage. This double mutant can be complemented by PLT1 or PLT3 when expressed under the PLT2 or PLT4 showing that expression domain rather than protein sequence plays a key role in diversification. It is a nice story that shines a new light on the control of meristematic potential during early embryogenesis. With the revisions the story now includes more focus on the function of PLT2 & BBM in the zygote and looks at PLT target gene expression across lineages.
Overall I think it is now more rounded story.

Comments for the author

I am in principle satisfied with the revisions made by the authors and I think the manuscript is suitable for publication.

After reading the comments from the other reviewer, my suggestion would be however, to link the author's findings more to other early embryonic regulatory mechanisms. Right now it is a PLT story

© 2024. Published by The Company of Biologists under the terms of the Creative Commons Attribution License (https://creativecommons.org/licenses/by/4.0/).

that happens to take place in the zygote/1-cell embryo which limits its scope. I think it would be valuable and exciting to connect it more to other zygote/1-cell embryo known mechanisms. For example the symmetrically dividing zygote in 3I/J is vaguely reminiscent of yda mutants. Consider expanding the discussion/introduction and placing these findings in the context of YDA/SSP signaling, WOX8/9 regulation, etc. Again, this is not essential but I think it would widen the article's scope and increase its relevance.

Reviewer 2

Advance summary and potential significance to field

The authors report the role of PLT genes in the early stage of embryo development.

This brings a new insight into PLT genes in controlling embryonic stem cells.

Comments for the author

The authors have addressed most of my concerns. I have no further suggestions.

© 2024. Published by The Company of Biologists under the terms of the Creative Commons Attribution License (https://creativecommons.org/licenses/by/4.0/).
